# Book Reviews

**Published:** 1892-08

**Authors:** 


### Book Reviews.

TRANSACTIONS OF THE SOUTHERN SURGICAL AND GYNECOLOGICAL ASSOCIATION. Vol. IV. Fourth session. Held at Richmond, Va., November 10, 11, 12, 1891. Edited by WILLIAM E. B. DAVIS, M. D., Secretary. Published by the Association. Philadelphia: Wm. J. Dornan, printer.

This volume is a record of the work done by this excellent association at its fourth annual meeting, held in Richmond, Va., in November, 1891, under the presidency of Dr. Lewis S. McMurtry, of Louisville. The book teems in interest from beginning to end, and it would be difficult to discriminate or to decide where to begin criticism, unless it be that criticism which culminates in praise.

The distinguished President chose for the subject of his address, A Plea for Progressive Surgery, in which he brings a terrible indictment against those so-called conservatives who, under the seductive mistake of conservative surgery, would condemn many women to a prolonged invalidism or else to woeful suffering, whose only termination can be in a slow but never-failing death. McMurtry starts out with the definition of the word "conservative," taken from Webster, which is, "Having power to preserve in a safe or entire state, or from loss, waste or injury." He protests against the perversion of the word "conservatism" from its true meaning, as given above, to that when it is applied, as it frequently is, to retard progress in surgery, and thus prevent that loss, waste, or injury from which it is expected to rescue the sufferer.

Dr. McMurtry has done the profession a great service in thus calling attention to the mistaken ideas and to the false teachings of the so-called conservatives. A surgeon is always conservative, in that he would not use the knife unnecessarily, but when men get up in medical meetings to announce the fact that they do not believe in this or that operation because someone has said that tyros have performed it unnecessarily, or because, perchance, some heretofore great leader changes front and announces himself an advocate of electricity, where previously he used the knife, it is time that the skilful abdominal surgeon, of which Dr. McMurtry is the type, shows up the distinction and contrasts the difference between a true conservatism, which would preserve from injury, loss, or death, by removing hopelessly diseased structures, and that false application of the term which seductively betrays into the very mire of disaster.

Dr. McMurtry deservedly castigated those men who in discussions flippantly allude to the "castration of women," to the "removal of ovaries," to "spaying," and to other improper terms, for the purpose of covering their own ignorance, and so planting themselves across the path of progress in the great movement towards improved pelvic surgery, by which, to use Dr. McMurtry's own words, hundreds and thousands of lives are saved.

When a diseased ovary or tube has passed beyond the reach of ordinary remedies that have been many times successful in their cure, when their structures have become hopelessly changed, and their functions destroyed, thereby threatening either life itself, or dooming the patient to prolonged or hopeless ill-health, there is no more striking example of true conservatism than in the surgeon that would at once remove these offending organs or structures.

The men who have carefully studied the anatomy and pathology of the pelvis and the abdomen, and have perfected themselves in the technique of pelvic and abdominal surgery, so that the records of their successes are very nearly one hundred per cent., are not to be shouted down by this dangerous misapplication of conservatism.

Among the more important papers read at this meeting may be enumerated the following: Albuminuria, its Relations to Surgical Operations, by J. W. Long, M. D., of Randelman, N. C.; Remarks on Systemic Infection from Gonorrheal Poisoning, by Bedford Brown, M. D., of Alexandria, Va.; Complications in Pelvic and Abdominal Surgery, and How to Deal with Them, by Joseph Price, M. D., of Philadelphia; Medico-Legal Aspect of Intestinal Surgery, by John D. S. Davis, M. D., Alabama; the Pedicle in Hysterectomy, by I. S. Stone, M. D., of Washington, D. C.; Injury to the Pelvic Floor, and the Method of Repairing the Same, by Thomas Addis Emmet, M. D., of New York; the Surgical Treatment of Anterior Displacement of the Uterus, by Charles A. L. Reed, M. D., of Cincinnati; the Part the Shoulders Play in Producing Laceration of the Perineum, with Suggestions for its Prevention, by W. D. Haggard, of Nashville; the Present Aspect of Cerebral Surgery, by Landon Carter Gray, M. D., of New York; the Venomous Reptiles of the United States, with the Treatment of Wounds Inflicted by Them, by Paul B. Barringer, M. D., of the University of Virginia.

The discussions on the several papers were of an interesting and instructive nature, and served to accentuate the teachings that the authors aimed to evolve in the text. The lights and shadows which strong discussions bring out are oftentimes useful in strengthening an author's position, as well as occasionally developing his weaknesses. In other words, such discussions serve to separate the chaff from the grain, and that has been most amply demonstrated in the volume before us. It is well illustrated with numerous wood-cuts and plates, while the printing and binding is in the best style of modern book art. Every progressive surgeon and gynecologist should subscribe to the annual volumes of this society.

---

A SYSTEM OF PRACTICAL THERAPEUTICS. Vol. III. Edited by HOBART AMORY HARE, M. D., Professor of Therapeutics and Materia Medica in the Jefferson Medical College of Philadelphia; assisted by WALTER CHRYSTIE, M. D. In three volumes, comprising 3,544 pages, with 434 illustrations. Per volume, cloth, $5.00; leather, $6.00; half Russia, $7.00. Sold by subscription only. Philadelphia: Lea Brothers & Co. 1892.

Following out the plan in our review notice of Volumes I. and II., by giving the contents, we here announce Volume III. in a like manner, the contents of which embrace the following subjects:

*Diseases of the Skin.*—Disorders of Secretion, by H. Radcliffe Crocker, M. D.; Inflammations of the Skin, by W. A. Hardaway; Hypertrophies and Atrophies of the Skin, by James Nevins Hyde, M. D.; Neuroses and Parasites of the Skin, by Milton B. Hartzell, M. D.

*Diseases of the Nervous System.* — Hospital Treatment of Insanity, by Edward N. Brush, M. D.; The Medical Treatment of Insanity, by H. M. Bannister, M. D.; Chorea, by B. Sachs, M. D.; Epilepsy and Tetanus, by J. Chalmers DaCosta, M. D.; Locomotor Ataxia, Acute Infantile Spinal Paralysis, Myelitis, and Amyotrophic Lateral Paralysis, by M. Allen Starr, M. D.; Apoplexy, Brain Tumor, Spinal Tumor, Meningitis, Cerebritis, and Neuritis, by Charles K. Mills, M. D.; Disorders of Sleep, by Landon Carter Gray, M. D.; Headaches and Neuralgia, by Wharton Sinkler, M. D.; Nervous Disorders and Paralysis from Excessive Use of the Parts Affected, Vertigo, Tremor, and Lead Poisoning, by Eugene Riggs, M. D.; Cerebral Concussion and Shock, by Joseph Ransohoff, M. D.; Morbid Habits, by T. D. Crothers, M. D.; Localized Spasms, Localized Palsies, Facial Hermiatrophy, by F. X. Dercum, M. D.

*Diseases of the Genito-Urinary Apparatus.* — Nephritis, Pyelitis, Phosphaturia, Chyluria, Albuminuria, Lithuria, Oxaluria, and Diabetes Insipidus, by Andrew H. Smith, M. D.; Gonorrhea and its Complications, Stricture, Cystitis, Hypertrophy of the Prostate, Atony of the Bladder, and Vesical Calculus, by J. William White, M. D.; Diseases of the Prepuce, Glans, Penis, and Testicles, by Edward Martin, M. D.; Diseases of the Vulva and Vagina (Non-Venereal), Leucorrhea, by T. J. Watkins, M. D.; Diseases of the Uterus, by R. L. Dickinson, M. D.; Amenorrhea, Dysmenorrhea, Menorrhagia, and Sterility, by Hunter Robb, M. D.; Diseases of the Broad Ligaments, Tubes and Ovaries, by Howard A. Kelly, M. D.; Diseases of Pregnancy, Parturition, and of the Puerperium, Extrauterine Pregnancy, and Abortion, by Barton Cooke Hirst, M. D.

*Diseases of the Eye.*—General Considerations of the Treatment of the Eye and its Disorders, by W. F. Mittendorf, M. D.; Diseases of the Conjunctiva, Sclera, and Cornea, by George E. DeSchweinitz, M. D.; Diseases of the Iris and Ciliary Body; Sympathetic Affections, by Charles J. Kipp, M. D.; Diseases of the Optic Nerve, Retina, Choroid, and Vitreus; Amblyopia and Amaurosis, by S. C. Ayres, M. D.; Diseases of the Lens and Glaucoma, by Swan M. Burnett, M. D.; Diseases of the Orbit, Lachrymal Apparatus and Eyelids, by Henry Graddle, M. D.; Diseases of the Ocular Muscles, Paralytic and Concomitant Squint, Asthenopia, Spasms of the Ocular Muscles, and Nystagmus, by Lucien Howe, M. D.; Optical Therapeutics; Normal and Abnormal Refraction; Presbyopia, Principles Involved in Fitting Glasses, by Edward Jackson, M. D.; Injuries of the Eye and its Appendages, by F. Buller, M. D.

*Diseases of the Ear.*—Diseases of the External Ear and Tympanic Membrane, by B. Alexander Randall, M. D.; Acute Diseases of the Middle Ear, by Robert Barclay, M. D.; Chronic Purulent Disease of the Middle Ear, by Samuel Sexton, M. D.; Chronic Catarrh of the Middle Ear, and Disease of the Internal Ear, by Charles H. Burnett, M. D.

The scope of this work is beyond that of any previous one on the subject. It is an evidence of great enterprise on the part of the publishers, and of stupendous energy and work on the part of the editor. Whatever may be said with reference to the value of perfection in the important departments of anatomy, physiology, and pathology, the goal, after all, is the treatment of disease, and work which contributes to its successful management is to be looked upon as of vast use to humanity. It cannot be denied that therapeutic resources, whether the treatment be confined to the mere administration of drugs, or allowed its more extended application to the management of disease, have so greatly multiplied within the last few years as to render previous treatises of little value. Herein will be found the great value of Hare's encyclopedic work, which groups together within a single series of volumes the most modern methods known in the management of diseases, and especially does it deal with important subjects comprehensively, which could not be done in a more limited treatise. This last volume will possess interest for the obstetrician and gynecologist where they will find, at least, two hundred pages devoted to these two departments. These pages are copiously illustrated, and add much to the interest in the work.

We cannot commend Hare's System of Practical Therapeutics too highly, because, even admitting what must always be admitted with reference to every such product, numerous imperfections, it still stands out first and foremost as a work to be consulted by authors, teachers, and physicians, throughout the world.

---

Treatise on the Diseases of Women, for the use of students and practitioners. By Alexander J. C. Skene, M. D., Professor of Gynecology in the Long Island College Hospital, Brooklyn, N. Y.; formerly Professor of Gynecology in the New York Post-Graduate Medical School; Gynecologist to the Long Island College Hospital; President of the American Gynecological Society, 1887; Corresponding Member of the British, Boston, and Detroit Gynecological Societies, of the Royal Society of the Medical and Natural Society of Brussels, and of the Leipzig Obstetrical Society; Fellow of the New York Academy of Medicine; Ex-President of the Medical Society of the County of Kings; Ex-President of the New York Obstetrical Society. Second edition, revised and enlarged, with 251 engravings and nine chromo-lithographs. New York: D. Appleton & Co. 1892.

When the first edition of this work appeared, we gave it an extended analytical review. (See Vol. XXVIII., page 345, January, 1889.) The second edition presents very little change in the make-up of the treatise or in the general arrangement of the text. New chapters have been added on Ectopic Gestation, Diseases and Injuries of the Ureters, Vesical Hernia and its Surgical Treatment, and the latest views of the author have been given in the discussion of Laparatomy, Ovaritis, and Injuries of the Cervix Uteri and Pelvic Floor.

The 251 wood engravings and nine chromo-lithographs serve to illustrate the text in a pointed and useful manner. Skene's treatise has become a text-book in a large number of the medical colleges of the land, hence a new edition has become necessary. Nothing has appeared from the press of late years that can be compared to it or compete with it in any way, except Pozzi's elaborate treatise lately issued. These two will serve to supply the needs for gynecological text-books for sometime to come, providing new editions are put forward with sufficient frequency to keep pace with the progress in the art. We have no doubt the enterprising publishers and the able authors of both these works will see to it that this is done. When we reviewed Skene's first edition, we accorded it the first place in text-book gynecological literature, and we are satisfied from comparison of the two editions that the author is laboring to keep it abreast of gynecological progress. It is a credit to the distinguished author, and a useful, if not necessary, volume for the specialist, teacher, and general practitioner.

---

INTERNATIONAL CLINICS: A Quarterly of Clinical Lectures on Medicine, Surgery, Gynecology, Pediatrics, Neurology, Dermatology, Laryngology, Ophthalmology, and Otology. By Professors and Lecturers in the leading medical colleges of the United States and Great Britain and Canada. Edited by JOHN M. KEATING, M. D., Philadelphia, Consulting Physician for Diseases of Women to St. Agnes' Hospital, Philadelphia; Editor Cyclopedia of the Diseases of Children.—J. P. Crozer Griffith, M. D., Philadelphia, Clinical Professor of Diseases of Children in the University of Pennsylvania; Professor of Clinical Medicine in the Philadelphia Polyclinic.—J. Mitchell Bruce, M. D., F. R. C. P., London, England, Physician and Lecturer on Therapeutics at the Charing Cross Hospital. And David W. Finlay, M. D., F. R. C. P., London, England, Physician to the Middlesex Hospital, and to the Royal Hospital for Diseases of Chest; Lecturer on Clinical Medicine in the Middlesex Hospital Medical School. Philadelphia: J. B. Lippincott Co. 1891.

The contents of Volume III. of this interesting and valuable periodical publication indicates its continued prosperity and improvement. Acute Intestinal Obstruction is discoursed upon by W. T. Gairdner, M. D., of Glasgow; Hodgkin's Disease, by Richard Lea Macdonald, M. D., of Montreal; Tubercular Peritonitis, by J. F. Goodhart, M. D., and George Carpenter, M. D., of London; Potts's Disease of the Spine, by V. P. Gibney, M. D., of New York; Tumors of the Bladder, by F. Sedgwick Watson, M. D., of Boston; Syphilis, by A. H. Ohman-Dumesnil, M. D., of St. Louis;

Abdominal Hysterectomy, by Henry T. Byford, M. D., of Chicago; Removal of Uterine Appendages, by F. H. Champneys, M. D., of London; Pyosalpinx, etc., by Clinton Cushing, M. D., of San Francisco; Lamellar Cataract, etc., by David Webster, M. D., of New York; Fibro-Sarcoma of the Naso-pharynx, by E. Fletcher Ingals, M. D., of Chicago; Vegetable Parasitic Disease of the Skin, etc., by Henry G. Piffard, M. D., of New York. These are only a few of the titles that we have selected for mention on account of their especial interest. They are taken at random from among a large group of interesting papers on a very wide range of subjects that fill this book. We have heretofore expressed our opinion as to the propriety of publishing this class of literature. We think it is an improvement over the voluminous text-books which contain so much and are read so little. The short monographs, which are called clinical lectures, published in the International Clinics, deserve and will receive careful examination at the hands of every physician who is interested in the several subjects of which they treat.

---

ENCYCLOPEDIA OF MEDICINE AND SURGERY. By various writers, enlarged, upon a new system, which embodies the methods of treatment employed by eminent practitioners of medicine. Compiled under the direction of the publishers, and including the writings of Drs. John Abercrombie, E. C. Beale, C. E. Beevor, Sydney Coupland, Frederick F. Eve, G. P. Field, J. K. Fowler, Wm. Gay, W. B. Hayden, G. E. Hermance, Victor Horsley, Henry Juler, C. B. Keetley, and others. New York: J. B. Flint & Co. 1892.

This large octavo, double-column volume of 738 pages contains a brief *résumé* of diseases and injuries, beginning with "Abdomen Injuries of the," and ending with "H." The general plan of the book is to give a concise definition of the practical subjects embracing the science of medicine and surgery, and particularly to give especial force and impact to the treatment of disease.

Publishers are constantly putting forth reference books that are of great value to the profession, and this one is no exception to the rule, but we question very much the continuation of this form of literature beyond the present lines laid down. There are so many of these reference works published that a library could easily be filled with them, to the exclusion of everything else. They tend to invite superficiality of research. The one before us is, as we intimated, of the better sort, and is easily worth the price it is offered at.

